# Child Crying in Utero

**Published:** 1876-09

**Authors:** W. H. Dean

**Affiliations:** Woodstock, Ga.


CHILD CRYING IN UTERO.—In the June number of the JOURNAL we published the report of a case of a "Child Crying in Utero," by Dr. W. H. Dean. In a foot-note we expressed regrets that he had not given a more detailed report of this very novel case. Some days ago we received the following note:

*Editors Atlanta Medical and Surgical Journal:*

In compliance with your published and written request, I give a more detailed account of "Child Crying in Utero."

Mrs. C. is about 30 years old; rather under medium size; pelvis well formed, but rather small; the mother of four children; has long, hard, and difficult labors, in the first of which she was delivered with forceps, attended with partial laceration of the perineum. Dr. Latimer was engaged and sent for in her last confinement, but being delayed, and the patient growing rapidly worse, I was also sent for, and arrived about twenty minutes after the rupture of the membranes and discharge of a large quantity of liquor amnii. Made an examination immediately; found the head high up, above the superior strait, and not engaged in the pelvis; os uteri well dilated and dilatable. There was a prolapsus of the cord and a right lateral obliquity of the uterus. No pains since the discharge of waters.

Placed her in the knee-breast position, and made an effort to return the cord, but the introduction of the hand caused so much pain, that, at her earnest entreaty, desisted for the time, intending to use every means within my knowledge to return it. She got up and sat in a chair while her bed was being arranged, and when she arose to return to bed, while standing on the floor, the child commenced crying vigorously, so as to be heard all over the room (14x16 ft.), and I think could have been heard all over the house (16x28 ft.), from floor to roof and from end to end. I was standing close beside the patient while the child was crying, and having heard the *first cry* of more than one thousand children, do not think I could be easily deceived. All the attendants heard it, and the patient appeared considerably agitated. About this time Dr. Latimer came in, and I turned over tho case to him to attend calls of my own.

The Doctor says: "The head was above the superior strait; made an effort to return the cord; failed and gave it up. About an hour after my arrival the child cried loud enough to be heard over a large room; after that cried again, but feebly. Two hours after my arrival the pains came on; in about thirty minutes the head engaged in the superior strait, left occipeto iliac position. It was not long until the cord ceased to pulsate, and after a hard labor of eight hours she was delivered of a large dead child. Convalescence slow; now well."

As to the case in its medico-legal aspects, or as to the great physiological question, whether a child breathing in utero could possibly live after a long, hard labor, I do not answer, but leave the discussion for others.

W. H. Dean, M.D.

*Woodstock, Ga., Aug* 28, 1876.

